# Application of Silica Nanoparticles for Improving Growth, Yield, and Enzymatic Antioxidant for the Hybrid Rice EHR1 Growing under Water Regime Conditions

**DOI:** 10.3390/ma14051150

**Published:** 2021-02-28

**Authors:** Omnia M. Elshayb, Abdelwahed M Nada, Heba M. Ibrahim, Heba E. Amin, Ayman M. Atta

**Affiliations:** 1 Rice Research and Training Center, Field Crops Research Institute, Agricultural Research Center (ARC), Giza 588, Egypt; omniaelshayb3434@yahoo.com (O.M.E.); nadaabdelwahed456@gmail.com (A.MN.); 2 Botany Department, Faculty of Agriculture, Mansoura University, Mansoura 35516, Egypt; hebaho@mans.edu.eg; 3 Department of Food Industry, Faculty of Agriculture, Mansoura University, Mansoura 35516, Egypt; heba_emad1127@hotmail.com; 4 Chemistry Department, College of Science, King Saud University, Riyadh 11451, Saudi Arabia

**Keywords:** agriculture domains, egyptian rice, physical characterization, silica nanoparticles

## Abstract

The current study was designed to assess the effect of different concentrations of silica oxide nanoparticles (SiO_2_NPs) (0, 30, 60, and 90 ppm) as foliar applications under three irrigation regimes i.e., irrigation every 3 days (IR3, control), irrigation every 6 days (IR6), and irrigation every 9 days (IR9) on growth, yield and certain metabolites of rice (*Oryza sativa* L. cv. EHR1). To achieve such a goal, 2 field experiments were conducted during the 2018 and 2019 seasons at the Experimental Farm of Rice Research and Training Center (RRTC), Sakha Agricultural Station, Kafr El-sheik, Egypt. Firstly, the as-prepared nanoparticles of SiO_2_ were prepared from useless materials (RHs) which are considered as one of the bio burdens on the environment via treating with HCl and followed by drying and calcination. Consequently, the synthesis was examined by making use of advanced tools such as X-ray diffraction (XRD), transmission electron microscopy (TEM), dynamic light scattering (DLS) for illustrating the hydrodynamic particle size of SiO_2_NPs and scanning electron microscopy (SEM). The nanoparticles were formed with nearly spherical shape and small size. The results indicated that leaf area index, dry matter production, the number of panicles/m^2^, the number of filled grains/ panicles, 1000 grain weight, grain yield, and biological yield as well as chlorophyll content have witnessed a significant increase under irrigated application every 3 and 6 days. Whilst a prolonged irrigation regime up to 9 days recorded a remarkable decline in the aforementioned characteristics except for the number of unfilled grains/panicle which increased considerably in both seasons. On the other hand, proline concentration and the activity of the antioxidant enzymes were increased in both irrigated treatments every 6 and 9 days compared with control treatment (irrigation every 3 days). The foliar supplementations of (SiO_2_NPs) contributed to ameliorating all the aforementioned characteristics progressively up to the dosage of 90 ppm compared to control treatment (no Si/NPS application) in both seasons. Invariably, growth and yield parameters in water-stressed plants treated with SiO_2_NPs were higher than those in water-stressed plants without SiO_2_NPs addition. Based on that, it could be concluded that the foliar application of SiO_2_NPs can mitigate the adverse effect of water stress on rice plants.

## 1. Introduction

Rice (*Oryza Sativa* L.) is a crucial cereal crop that supplies around half of the Earth’s people within 6% of the daily calorie intake [1]. It is worth noting that to meet global needs in 2035, rice productivity must be increased by 26% [2]. Various drawbacks restrict the yield of the rice crop and have a devastating impact on rice cultivation including pest infestation, unfavorable environmental conditions accompanied by climate change, salinity stress, insufficient nutrients, and deficit irrigation (DI) as a result of an emerging shortage of water resources. The annual rice production shortfall due to DI approximated 18 million tons [2,3]. In comparison with other cereals, the role of combating and diluting the adverse effect of DI is much more complex in rice due to its higher sensitivity to DI [4]. The adverse impact of DI on rice was manifested by reducing root volume, plant height, leaf dimension, leaf greenness, tillering, panicle exertion, flowering, and fertility, all of which have a negative impact reflected in yield loss [5,6]. Moreover, DI resulted in chlorophyll breakdown and enhanced generation of reactive oxygen species (ROS), which attack cell biological components, thereby affecting adversely and in a deadly way the integrity and functions of plant cells [7]. Correspondingly, the influence of water-conserving water irrigation regimes mostly on the growth and yield of *Oryza sativa* L. cv. EHR1 should be examined. Silicon is a major contributor to the Earth’s crust after oxygen. It is not considered a vital nutrient for most terrestrial plants, but it plays an important role in improving the quality, quantity, and protection of some plants, especially rice and wheat. It also has the ability for providing resistance towards harsh conditions in plants [8]. The reduction of silicon-induced stress as mentioned in previous studies is attributed to structural as well as biochemical effects, such as enhancing photosynthetic parameters [9], improving activities of antioxidant enzymes as well as increasing soluble protein content, and preserved water relations of the plant cells [10].

The utilization of nanomaterials has produced several notable results in improving growth, yield production, and alleviate the influence of different stresses on plants. Among elegant and unique nanomaterials, silica nanoparticles (SiO_2_NPs) which have benign efficacy in remediating the negative effect of heavy metals [11,12], salinity [13], ultraviolet (UV-B) radiation [14], pathogens, and insects [15,16,17] was reported. Besides, the drought stress-mitigation effects of SiO_2_NPs on cereals plants are well-documented [18,19,20]. Nevertheless, its drought stress mitigation effectiveness on a semi-hydrophyte like rice is less investigated and poorly understood. Various effects were reported as the basis of SiO_2_NP-induced alleviation of drought stress. Nanoparticles of SiO_2_ at 400 mg/L, 2000 mg/L, and 4000 mg/L increased the content of photosynthetic pigments (chlorophyll a, b and carotenoids) in *Z. mays* compared with control [21]. Furthermore, the application of SiO_2_NPs caused a significant increase in the content of soluble sugar and the activities of catalase (CAT) and peroxidase (POD) in faba bean leaves [22]. Whether growth, yield, and stress-related metabolites in a high water-demanding crop like rice will be similarly affected by SiO_2_NPs fortification needs to be ascertained. This investigation was conducted to assess the effect of silica nanoparticles (SiO_2_NPs) at different concentrations (30 ppm, 60 ppm, and 90 ppm) on growth, yield, and some key stress-related metabolites of the Egyptian hybrid rice variety EHR1 plants subjected to moderate and severe water stress. The work was aimed to prepare the valuable SiO_2_NPs from useless wastes aiding to remove one of the issues that cause problems for the environment. Moreover, the prepared nanoparticles can be scaled up on a large scale without any noticeable cost. 

## 2. Experimental Details

### 2.1. Synthesis of Silica Nanoparticles (SiO_2_NPs) from Useless Materials (RH Silica)

Silica nanoparticles were prepared after useless materials (RHs) were treated with HCl, then calcination. In brief, running tap water was used to wash RHs, then washing with deionized water was important to remove any dirt or other contaminants stacked on RHs. The RHs were dried at 90 °C along the night. After drying, the portion of RHs was refluxed with 5 wt.% HCl solution for 2 h, then washed with deionized water and dried at 90 °C for at least 12 h. The produced SiO_2_NP with a diameter of ca. 60−70 nm was obtained by calcining HCl-treated RHs in a furnace at 700 °C for 2 h. The yield of SiO_2_NPs was about 17 wt.%. Then the as-synthesized SiO_2_NP was characterized as follows: The SiO_2_NPs sample was deposited on a carbon-coated copper grid and left for drying at room temperature to be examined with a transmission electron microscope (TEM, JEOL 200 kV, Jeol, Tokyo, Japan). Selected area diffraction (SAED) was utilized to investigate the nature of the prepared nanoparticles in terms of their amorphous state. For the evaluation of the particle size and size distribution of the as-prepared SiO_2_NPs, dynamic light scattering (DLS, Nano-Sizer SZ90, Malvern instruments Ltd., Malvern, Worcestershire, UK) was used at pH = 7 and 25 °C. Scanning electron microscopy was scanned using a field emission scanning electron microscope (FESEM, ZEISS GeminiSEM 360; Mladá Boleslav, Czech Republic). X-ray diffraction pattern (XRD-X’Pert Pro, PANalytical, Almelo, The Netherlands) was used to investigate the crystalline lattice structure.

### 2.2. Cultivation and Experimental Conditions

Two field experiments were conducted during the two cropping seasons of 2018 and 2019 at the Experimental Farm of Rice Research and Training Center (RRTC), Sakha, Kafr-Elsheikh, Egypt. The previous cultivated crop was wheat during both seasons of the study. Egyptian hybrid rice (EHR1) variety was used in this study. Pregerminated seeds (healthy hybrid rice grains at the rate of 24 kg/ha were soaked more than water for 24 h and further incubated for another 48 h to enhance germination) were broadcasted in the nursery on 11 and 14 of May in the 2018 and 2019 seasons, respectively. Three seedlings per hill at 25 days old were transplanted in the permanent field’s experimental plots at a 20 cm × 20 cm distance between hills and rows in 15 m^2^ (5 m × 3 m) size plots. Weeds were controlled chemically using Saturn 50% at the rate of 5 L/ha at five days after transplant. Nitrogen in the form of urea (46% N) at the rate of 165 kg/ha was applied as recommended in two doses; 2/3 basal application + 1/3 at panicle initiation. The recommended phosphorous and Potassium fertilizers in the form of calcium superphosphate (15% P_2_O_5_) at a rate of 37 kg P_2_O_5_/ha and potassium sulfate (48% K_2_O) at the rate of 50 kg K_2_O kg/ha were applied. Zinc fertilizer, applied at the rate of 24 kg/ha ZnSO_4_, was mixed with sand, and manually broadcasted before transplanting. During both seasons, representative soil samples were taken from the experimental site at the depth of 0–30 cm. The physical and chemical characteristics of the experimental soil were analyzed according to [23] and presented in Table 1.

The experiment was laid out in a split-plot design with four replicates. The main plots were devoted to three water regimes; irrigation every 3, 6, and 9 days which denoted as IR3, IR6_,_ and IR9 respectively. The subplots were assigned to four treatments of SiO_2_NPs namely, SiO_2_NPs 0, control; SiO_2_NPs 30, foliar application of SiO_2_NPs at the rate of 30 ppm; SiO_2_NPs 60, foliar application of SiO_2_NPs at the rate of 60 ppm and SiO_2_NPs-90, foliar application of SiO_2_NPs at the rate of 90 ppm. In the SiO_2_NPs 0 treatment, the foliar spray was done with distilled water. Treatments of SiO_2_NPs had been applied thrice, at mid-tillering, panicle initiation, and full heading. Main plots containing irrigation treatments were tightly separated by ditches, 2 m wide and 1 m depth. The irrigation regime treatments, applied as mentioned above, were commenced after 10 days from transplanting.

### 2.3. Studied Traits

#### 2.3.1. Plant Growth Characteristics

At the heading stage, plants of five hills were randomly taken from each plot to estimate leaf area index (LAI) and dry matter production. Leaf area index is the ratio between the leaves areas (cm^2^) of the plant divided by the ground area occupied by the plant. Dry matter production (g/m^2^) was estimated as described by [24].

At the harvest stage, panicles of five random hills from each plot were counted then converted to the number of panicles/m^2^. Ten panicles were randomly collected from each plot to determine the number of filled grains/panicles, and unfilled grains/panicle and 1000 grain weight (g). The biological yield (both grain and strawweight) was measured from an area of 12 m^2^ (3 m × 4 m) which was harvested from each plot at random avoiding the border effects. Grain yield was adjusted to 14% moisture content as described by [25].

#### 2.3.2. Determination of Chlorophyll and Proline Content

Chlorophyll content: total chlorophyll content was determined as SPAD (Soil Plant Analysis Development) value in ten flag leaves using chlorophyll meter (Model–SPAD 502) Minolta, Japan.

Determination of proline: proline concentration was determined according to the method of [26]. About 300 mg of leaf tissue was homogenized in 10 mL of 3% (*w*/*v*) aqueous sulfosalicylic acid and filtered. To 2 mL of the filtrate was added 2 mL of ninhydrin acid, then 2 mL of glacial acetic acid was added, and the mixture was boiled for 60 min. The mixture was extracted with toluene and the free proline was quantified by spectrophotometry at 520 nm.

#### 2.3.3. Determination of the Activity of the Antioxidant Enzymes

Leaf tissues were homogenized (1:5 *w*/*v*) in an ice mortar using 50 mM sodium phosphate buffer, pH 7.0, containing 1 M NaCl, 1% polyvinyl pyrrolidone and 1 mM ethylenediaminetriacetic acid (EDTA). After centrifugation (20,000× *g*, 15 min), the supernatant (crude leaf extract) was used to determine enzyme activity; and hereinafter referred to as the enzymatic extract (EE).

CAT activity (EC 1.11.1.6) was evaluated by the decomposition of H_2_O_2_ according to [27]. At 200 µL, 1 aliquot of EE was added to 1.8 mL of the reaction mixture (RM) containing 50 mM K-P buffer (pH 7.0) and 30 mM H_2_O_2_. The decrease in H_2_O_2_ was accompanied by a decrease in absorbance at 240 nm. One unit of CAT activity is defined as the amount of enzyme that breaks down 1 M H_2_O_2_ in one minute.

The POD activity (EC 1.11.1.7) was analyzed according to the method [28] by adding 25 μL of EE to 2 mL of a solution containing 50 mM of K-P buffer (pH 6.8), 20 mM of guaiacol, and 20 mM H_2_O_2_. After 10 min of incubation, the reaction was stopped by adding 0.5 mL of 5% (*v*/*v*) H_2_SO_4,_ and the optical density was measured at 480 nm. One unit of POD activity is defined as the amount of substrate transformed by the enzyme in 1 min.

The SOD activity (EC 1.15.1.1) was analyzed according to the method of Van [29]. An aliquot of 50 μL of the enzyme extract was mixed with a solution containing L-methionine (13 mM), nitroblue tetrazolium chloride (75 μM), EDTA (100 μM) and riboflavin (2 μM) in a 50 mM potassium-phosphate-buffer (pH 7.8). The assay was performed in a chamber illuminated with 30 W fluorescent lamps. The reaction was started by turning the lamp on and terminated 5 min later by turning it off. The substrate formed as a result of NBT’s (nitro blue tetrazolium) photoreduction (the blue formazan) was estimated as the increase in absorption at 560 nm. The control reaction mixture had no enzyme extract. The blank solution contained the same constituents included in the complete reaction mixture but was kept in the dark. The amount of enzyme required to inhibit 50% of the NBT photoreduction in comparison with tubes lacking the enzyme extract was considered as one unit of SOD activity.

### 2.4. Statistical Analysis

All statistical analyses used analysis of variance technique employing the “COSTATC” computer software package. Treatment means were compared by Duncan’s Multiple Range Test [30].

## 3. Results and Discussion

The first target of our current research work was designed to achieve two promising goals. The first one is to prepare SiO_2_NP from useless materials and that caused a hazardous effect on the environment. This target was initially characterized using TEM, SEM, DLS for determining the hydrodynamic particle size, and XRD. As mentioned in the experiment part for the preparation of SiO_2_NPs, the process for the preparation is very cost-effective and no need for extra chemical or advanced instruments for preparation, just washing, drying, and calcination, which, in turn, is a promising method to be called up to obtain large production of SiO_2_NPs suitable for many huge industrial and agricultural domains. Then, the characterized SiO_2_NP was used as a foliar treatment at different concentrations (30, 60 and 90 ppm) as will be discussed in the second part of our current research work. Below are the characteristics data that confirm the preparation of SiO_2_NPs.

### 3.1. Utilized Advanced Tools in Terms of Transmission Electron Microscopy (TEM), Dynamic Light Scattering (DLS), Selected Area Diffraction (SAED), Scanning Electron Microscopy (SEM), and X-ray Diffraction (XRD) for Affirming the Preparation of Silica Oxide Nanoparticles (SiO_2_NPs)

**Figure 1:**
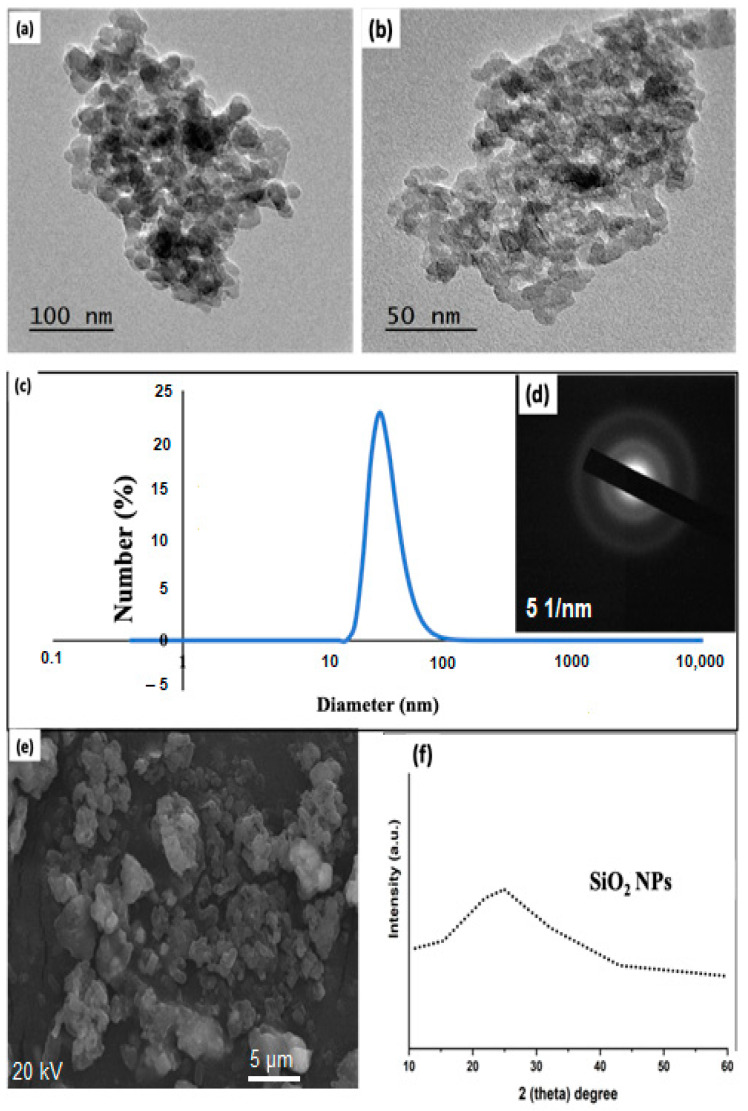
Transmission electron microscopy (TEM) of SiO_2_NPs at different magnification (**a**,**b**), (**c**) average hydrodynamic size, (**d**) selected area diffraction (SAED), (**e**) scanning electron microscopy (SEM) and (**f**) X-ray diffraction (XRD) of silica oxide nanoparticles (SiO_2_NPs).

First of all, to describe the shape and prediction of the particle size of the resultant SiO_2_NPs, TEM was used. SiO_2_NPs (Figure 1a) has appeared clearly spherical and nearly as uniform particle size. It is noted that the spherical particle’s average diameter is about 30 nm. For clarification of the particle shape and size distribution of SiO_2_NPs, the image (Figure 1b) is taken at high magnification. It can be assumed that SiO_2_NPs have a structure with a spherical shape. The insignificant agglomeration for these very small particles could be attributed to the absence of a stabilizing agent and the high surface area which facilitates the tendency of particles to be agglomerated in cluster form. However, these cluster agglomerated particles are still in small size and do not exceed 30 nm in all conditions of agglomerations.

Next, hydrodynamic average particle size is very important to be measured using DLS to stand on the actual size of the prepared SiO_2_NPs. The obtained particle size from DLS is set in Figure 1c. Note that the average particle size is 43 nm. As observed in Figure 1c, the image contains one sharp peak which confirmed the particles are formed in the monodisperse form with a polydispersity index equal to 0.107. The difference in the formed diameter between and TEM and DLS could be related to the swelling properties of SiO_2_NPs in a solution. As known, DLS needs during the measurement that the particles are dispersed in water and the analysis was carried for more than 18 times. All these runs make these particles swell and form a large particle. Nonetheless, the particle evaluated by both TEM and DLS is still below 50 nm which confirmed the small size and the large surface area (active materials) of these nanoparticles that are expected to have a very important role and efficiency in the agriculture field as will be presented in the second target of our current research work. Returning to our discussion and for further confirmation, the SAED pattern was conducted (Figure 1d). As clearly remarkable that, the particles are formed in the amorphous state with no spots or light points which affirms that the calcination step is very critical in our work to convert the useless compound into nanoparticles of SiO_2_ in nanoparticle form. The surface structure and morphology of SiO_2_NPs was further investigated using SEM. Figure 1e displays the morphological structure of SiO_2_NPs. SiO_2_NPs as fairly uniform spherical particles but the particles are remarkably formed with sufficient aggregation which could be attributed to the absence of the extra stabilizing agent. Therefore, spherical SiO_2_NPs are formed due to isotropic growth. Briefly speaking, the anisotropic structures of SiO_2_NPs could be induced by growth rates in different directions and as such, due to isotropic growth, nearly spherical SiO_2_NPs are formed. In brief, the calcination step (high temperatures) has resulted in the development of this shape (nearly spherical particles) which has led to extreme anisotropic growth, leading to the formation of aggregated particles due to the collision between a particle that causes the production of large particles at high temperature. Ultimately, the data of XRD (Figure 1f) agree with the data of SAED in which SiO_2_NPs in the amorphous state have no peaks for the crystallinity.

### 3.2. Application of SiO_2_NPs as a Foliar for Rice Plants

#### 3.2.1. Plant Growth Characteristics

Compared with IR3 (normal irrigation, no stress) leaf area index (LAI) and dry matter (DM) accumulation in plants irrigated every 9 days (IR9 plants) are decreased whereas those parameters, in plants irrigated every 6 days (IR6 plants), are not significantly affected in both seasons (Table 2). On the other hand, SiO_2_NPs with different concentrations increased both LAI and DM accumulation, in a concentration-dependent manner, i.e., both parameters are increased with increasing SiO_2_NPs concentration. Foliar spray of SiO_2_NPs at 90 ppm increased the leaf area index by 8.1% and 9.7%, and dry matter production by 6.0% and 6.5% in both the first and second seasons, respectively. The effects of both IRs and SiO_2_NPs were consistent during the two experimental seasons. The interaction effect between IRs and SiO_2_NPs levels on LAI was significant only during the first experimental season whereas the corresponding effect on DM was significant only during the second experimental season. During the experimental season of 2018, the highest LAI in water-stressed plants was recorded in plants that were irrigated every 6 days and treated with SiO_2_NPs at 90 ppm (IR6-SiO_2_NPs-90 plants). Moreover, in IR6 plants, there was no significant difference between LAI in SiO_2_NPs-0 plants and either SiO_2_NPs-30 or SiO_2_NPs-60 plants. Noticeably, SiO_2_NPs treatments did not affect LAI in IR9 plants (Table 3). Considering the impact of the interaction between the two experimental factors on DM during the experimental season of 2019 (Table 4), it is obvious that there was a dramatic increase in DM only attributable to the treatment of SiO_2_NPs at 90 ppm in IR3 plants, owing to both treatments of SiO_2_NPs at 60 ppm and 90 ppm in IR6 plants, and according to all concentrations of SiO_2_NPs in IR9 plants, with the highest adopted SiO_2_NPs level (90 ppm) being the most effective treatment in this regard.

Water stress-induced growth inhibition recorded in the current research work is in line with the findings of [31]. Water shortage is linked with nutrients malabsorption especially K^+^ which has a tremendous role in the water relations of plant cells [32]. Subsequently, losses in leaf chlorophyll, defects in both cell division and enlargement have been accrued thereby hindering dry matter production. Moreover, the generation and accumulation of abscisic acid (ABA) in the plant cell resulted in stomatal closure, decrement of both gas exchange, and CO_2_ assimilation which hurts the cell physiological pathway [19]. Contrariwise, treatment with SiO_2_NPs rendered a positive response in the estimated growth-indicative parameters, with the superiority of the higher adopted level (90 ppm). In conformity with the results of the present study, [33] demonstrated that Si enhanced both root and shoot biomass of rice under either normal or stressed conditions. Similar results are reported in wheat [18] and barley [20]. In this context, [9] described the benefits of silicon to rice plant tissues. He explained that Si deposits in the cell wall of rice leaves and stems, resulting in erected leaves especially under mutual shading. Accordingly, rice plants probably take higher advantage of light interception, thereby activating photosynthetic rate, enhancing chlorophyll content, leaf area index, and dry matter production. As indicated by [34], Si-induced cytokinin synthesis and may be reflected in increased chlorophyll content and an enhanced photosynthesis process.

#### 3.2.2. Yield and Yield Attributes

Data in Table 5 and Table 6 reveal significant and different responses on rice yield attributes (the number of panicles/m^2^, the number of filled grains/panicles, and unfilled grains/panicle and 1000 grain weight) as well as on both grain and biological yield under various treatments. The imposition of DI reduced all recorded yield parameters of rice whereas the number of unfilled grains is significantly increased, proportionately with increasing the stress magnitude. Nevertheless, the decrease recorded in all the estimated yield components in IR6 plants is not significant. These results are consistent during both experimental seasons. In IR9 plants (irrigated every 9 days), the number of panicles/m^2^ is decreased by 27.7% and 28.4%; the number of filled grains/panicles is decreased by 22.9 and 24.4%; 1000 grain weight is decreased by 5.9% and 6.2 %; grain yield is decreased by 27.3% and 27.4% and biological yield is decreased by 20.3% and 20.7% in the first and second experimental season, respectively, relative to the values in IR3 plants. The aforementioned data are supported by those reported by [31,33]. The reduction in the number of panicles/m^2^ and the number of filled grains/panicle in IR9 plants may be due to decreased chlorophyll content and reduced leaf area, and as a consequence disrupted leaf carbohydrate metabolism, thereby reduced assimilates transported to the sink organ (grains) and increasing reproductive abortion [31]. In this regard, it was stated that DI caused a marked decline in nutrient uptake and, consequently, the plant became under nourished, which probably decreased reproductive tillers, number of filled grains/panicle, and grain weight and grain yield outcome [32].

Concerning SiO_2_NPs effects, both yield attributes and grain yield increased progressively until the highest adopted level, i.e., to the treatment of SiO_2_NPs-90 (Table 5 and Table 6). Relative to control treatment (SiO_2_NPs-0), treatment with SiO_2_NPs at 90 ppm increased the number of panicles/m^2^ by 7.4% and 8.4%, the number of filled grains/ panicle by 5.8 and 6.6%, 1000-grain weight by 2.4% and 2.6%, grain yield by 8.4% and 9.0% and biological yield by 9.5% and 10.0% whereas unfilled grains/panicle decreased by 22.9% and 22.8% in the first and second experimental season, respectively. Si-induced water stress mitigation was explained by indicating that Si involves cell signaling because it has an immense function in binding the hydroxyl group of proteins [34,35]. Under DI conditions, silicic acid (the form in which the plant uptake Si) is polymerized and converted to silica gel. Silica gel concentrated on the surface of shoot parts as a double layer causing a remarkable decrease in water loss by leaf transpiration [36,37].

Induction of rice fertility, which is reflected in enhancing yield, in response to SiO_2_NPs, has been previously reported [18]. Furthermore, [38] described that the addition of SiO_2_NPs at the rate of 50 ppm at early tillering, mid tillering, panicle initiation, and full heading caused a significant increase in fertile tillers/hill, filled grain per panicle, grain yield, and straw yield compared with control. The beneficial effect of SiO_2_NPs on grain yield may be ascribed to enhancing panicle fertility during grain filling [39]. Moreover, it is suggested that supplementation of Si in the nano form resulted in elevating Zn content in plant tissues, which lead to enhanced productivity [40]. It is worth noting that Si causes amendments of C/N homeostasis by remobilization of amino acids manifested by an enhancement of N needs during grain development [41].

The interactive treatments between IRs and SiO_2_NPs concentrations significantly affected the number of panicles/m^2^ and 1000 grain weight, as well as both grain and biological yield in both seasons (Table 7, Table 8, Table 9 and Table 10). The highest values for all these parameters are recorded in IR3 plants that are treated with SiO_2_NPs at 90 ppm, whereas the lowest is recorded in IR9 plants that are not treated with SiO_2_NPs in both experimental seasons. However, IR9 plants treated with SiO_2_NPs at 90 ppm gave an increase in grain productivity by 7.4% and 10.8% compared to IR9 plants with no SiO_2_NPs application. All these parameters in IR6 plants that are treated with SiO_2_NPs at 90 ppm are not significantly different than those in IR3 plants which are treated with the same level of SiO_2_NPs.

The positive impact of SiO_2_NPs supplementation on the yield of water-stressed plants, as noted in the current study, is in line with the findings of [18] who recorded a 25% and 17.81% increase in wheat grain yield of water-stressed plants that are treated with SiO_2_NPs as foliar and soil application, respectively. It should be noted that [12] claimed that supplementation with SiO_2_NPs at 100 mg kg^−1^ enhanced biomass and yield outcome of wheat plants grown under two deficit irrigation regimes; 70% and 35% of water-holding capacity. Application of SiO_2_NPs alleviated water stress resulted not only from drought but also from salinity stress. In salt-stressed rice, the application of SiO_2_NPs at the rate of 25 mgL^−1^ enhanced the number of grains/panicle, panicle length, and 1000 grain weight as well as both grain and straw yield [42]. In this regard, it has been reported that the treatment with SiO_2_NPs caused a noticeable increase in leaf lignin content and net C assimilation of rice plants, which reinforces the plants for combating against different harsh stresses [16].

#### 3.2.3. Effects of Water Regimes, SiO_2_NPs Concentrations, and Their Interaction on Chlorophyll and Proline Content

In comparison with IR3 plants, chlorophyll content, determined as SPAD value, is decreased in IR9 plants but did not significantly affect IR6 plants (Table 11). On the other hand, all tested SiO_2_NPsconcentrations increased chlorophyll content in a concentration-dependent manner. The effect of the interaction between IRs and SiO_2_NPsconcentrations is significant during both experimental seasons (Table 12). During both seasons, the highest chlorophyll content is recorded in the leaves of IR3 X SiO_2_NPs-90 plants, whereas the lowest is recorded in those of IR9 X SiO_2_NPs plants.

The depressive effect of water stress on chlorophyll content is recorded in the current study and has also been previously reported by [20,31]. Additionally, [18] documented an increase in chlorophyll concentration under various stressful water regimes when the wheat plants have been treated with SiO_2_NPs using different concentrations (30, 60, and 90 ppm) under various stressful water regimes. Since the production of reactive oxygen species is mostly caused by excess energy absorption in the photosynthetic apparatus, such an issue can be prevented by destroying or degrading the absorbing pigments [43]. Moreover, water stress-induced K^+^ deficiency may cause losses in leaf chlorophyll [32]. Also, in alignment with the gained results of the current research study [20] reported an increase in chlorophyll and carotenoid content in barely seedling in response to plants treated with SiO_2_NPs at the rate of 125 mL/L in moderate conditions of water-stress.

**Figure 2:**
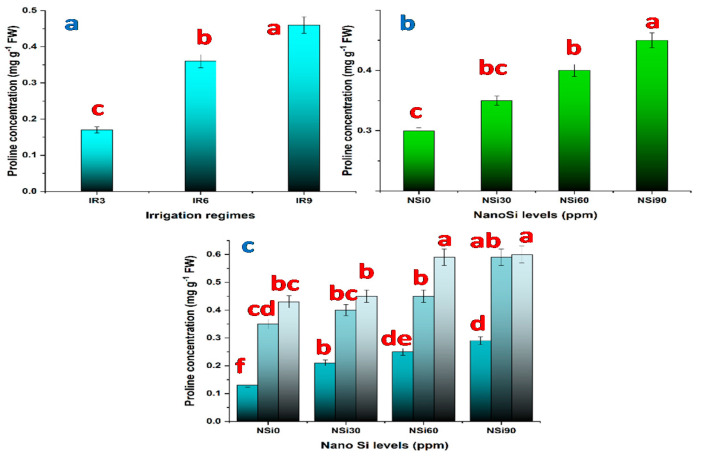
Proline concentration in rice leaves as affected by (**a**) irrigation regimes, (**b**) nano Si levels, and (**c**) their interactions.

On the other hand, proline concentration is increased significantly under water-stressed plants compared with unstressed plants (Figure 2a). Relative to the concentration in IR3 (unstressed plants), proline concentration is increased by 86.9% and 117.3% in response to IR6 and IR9, respectively. Moreover, SiO_2_NPs treatments increased proline concentration; however, the increase recorded in response to 30 ppm SiO_2_NPs is insignificant (Figure 2b). At 60, 90 ppm SiO_2_NPs, proline concentration increased by 33.3%, 56.6%, respectively. The interaction between IRs and SiO_2_NPs levels is significant. The highest proline concentration is recorded in plants that are irrigated every 9 days and treated with SiO_2_NPs at 90 ppm, followed by that in plants exposed to the same level of water stress and treated with SiO_2_NPs at 60 ppm (Figure 2c).

The response of plants to drought stress involves several changes in physiology and metabolism that displayed remarkable differences in their morphology. The result of the present study implied that deficit irrigation increased proline content in rice plants (Figure 2a). Following the findings of our current study that the content of proline increased in response to water stress in rice. Besides that, the content of proline for all upland rice varieties increases dramatically to the highest drought level [44]. Proline accumulation in plants under water stress protects the cell by balancing the osmotic potential of cytosol with that of vacuole and the external environment [45]. Accumulated proline could, therefore, serve as a compatible solute that controls and reduces plant cell water loss during water deficit and plays an important role in osmotic balance [46]. Moreover, accumulated proline under stress also provides energy for survival and growth, thereby, enabling the plants to resist stress conditions with regard to these phenomena, proline content is considered a good indicator for screening drought-tolerant genotypes in water stressful environments [4,47].

#### 3.2.4. Antioxidant Enzymes Activity

**Figure 3:**
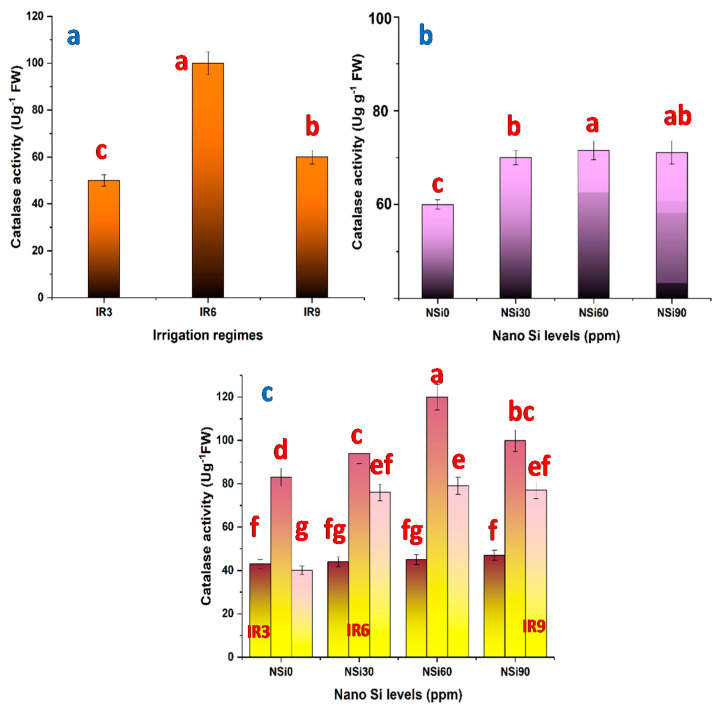
Catalase activity in rice leaves as affected by (**a**) irrigation regimes, (**b**) nano Si levels, and (**c**) their interactions.

**Figure 4:**
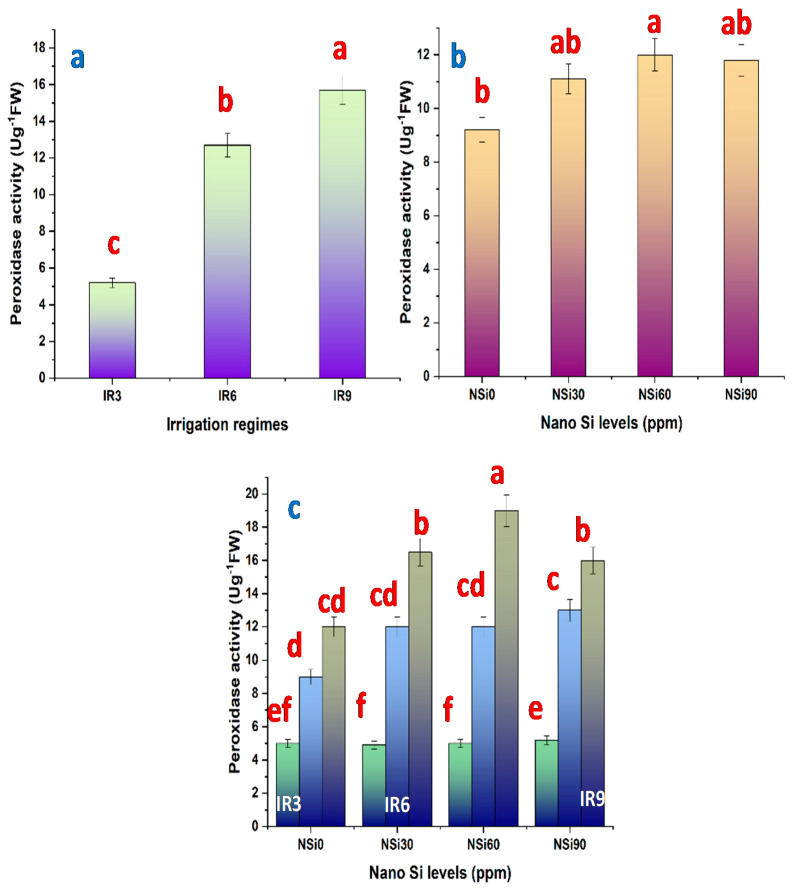
Peroxidase activity in rice leaves as affected by (**a**) irrigation regimes, (**b**) nano Si levels, and (**c**) their interactions.

**Figure 5:**
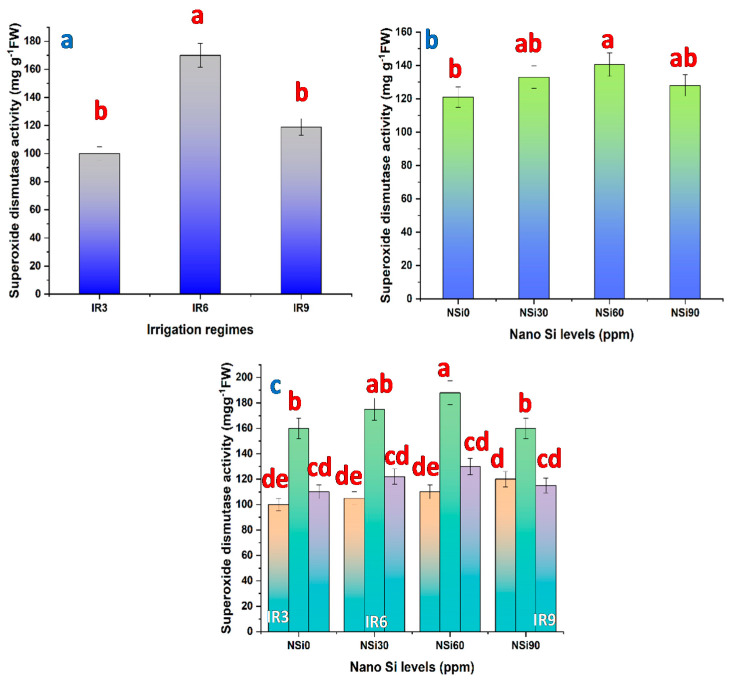
Superoxide dismutase in rice leaves as affected by (**a**) irrigation regimes, (**b**) nano Si levels, and (**c**) their interactions.

Prolonging the irrigation interval from 3 to 6 days increased the activity of CAT (Figure 3a), POD (Figure 4a), and SOD (Figure 5a). With increasing the magnitude of water stress further by extending the irrigation interval to every 9 days (IR9), the activity of POD tended to be increased further, whereas those of CAT and SOD tended to be decreased, but still higher compared with control plants. However, the increase recorded regarding SOD activity in IR9 plants was not significant. Compared with the activity of POD in control plants, this activity was increased by 129.4% and 211.7% in IR6- and IR9-plants, respectively.

All levels of SiO_2_NPs significantly increased the activity of CAT (Figure 3b), and the highest increase was recorded in response to SiO_2_NPs at 60 ppm. Although all tested levels of SiO_2_NPs increased the activity of POD (Figure 4b) and SOD (Figure 5b) compared with control plants, the increase was significant only in plants treated with SiO_2_NPs at 60 ppm. So, when plants were treated with SiO_2_NPs at 30 ppm, the activity of both POD and SOD was elevated, and further increased when the level of SiO_2_NPs was increased to 60 ppm, then decreased by increasing the level of SiO_2_NPs to 90 ppm.

The interaction between irrigation intervals and SiO_2_NPs levels affected significantly the activity of CAT (Figure 3c), POD (Figure 4c), and SOD (Figure 5c). The highest increase in CAT and SOD activity was recorded in plants irrigated every 6 days and treated with SiO2NPs at 60 ppm. On the other hand, the highest POD activity was recorded in plants irrigated every 9 days and treated with SiO_2_NPs at 60 ppm.

Drought stress contributes to the oxidative stress induced by the accumulation of reactive oxygen species (ROS), created mostly in chloroplast and to some extent in mitochondria. Plants could eventually create various types of antioxidants to detoxify ROS that reduces oxidative damage and confer resistance to drought. The ROS scavengers may be enzymatic including superoxide dismutase, peroxidase, and catalase, or non-enzymatic e.g., ascorbate, glutathione, and tocopherols. By extending irrigation duration, from “every three days” to “every six days”, the activities of the antioxidant enzymes, CAT, POD, SOD are increased (Figure 3, Figure 4 and Figure 5).

In water-stressed rice, higher ROS-scavenging enzyme activities have been identified and it has been implied that in water-stressed that the antioxidant system’s component plays a key role in plant protection against water stress rice [48]. In compliance with [49], since plants have established enzymatic and non-enzymatic mechanisms to scavenge ROS, sensitivity to drought-stress in plants corresponds to the levels of antioxidant systems and the substrate is higher, which implies that plants will produce more CAT, SOD, and POD under conditions of drought to remove the extra ROS in cells. Catalase has the potential to directly dismutase H_2_O_2_ into H_2_O and O_2_ and is indispensable for ROS detoxification in peroxisomes during stress conditions [50]. Superoxide dismutase detoxifies superoxide anion (O_2_^−^) by forming H_2_O_2_, and then the H_2_O_2_ can be eliminated by CA and POD [51]. Moreover, POD is also involved in various plant processes, including lignification [52], oxidation of phenolics [53], regulation of cell elongation, and detoxification of toxic compounds such as H_2_O_2_ [54]. Additionally, stress-tolerant genotypes have been distinguished by the higher activities of antioxidant enzymes. In particular, the drought-tolerant genotypes of *Triticum aestivum* [51,55] have higher activities of SOD, POD, and CAT than the drought-sensitive species.

According to the results of the present study, SiO_2_NPs increased proline content and enhanced the activity of the antioxidant enzymes in a concentration-dependent manner (Figure 2, Figure 3, Figure 4 and Figure 5). Similar results are reported with regard to proline and antioxidant enzymes. It seems that SiO_2_NPs could alter the activity of antioxidative enzymes in plant organs to improve stress tolerance, suggesting that the treated plants possess a better scavenging ability [18,20,48]. Moreover, it has been reported that the highest activities of superoxide dismutase, catalase, and peroxidase in Changbai larch (*Larix olgensis*) and soybean plants are recorded in response to SiO_2_NPs [56].

## 4. Conclusions

It could be concluded based on the results obtained that the Egyptian rice cultivar EHR1 is not highly water-demanding so that irrigation every 6 days did not cause harm either to its growth or to its yield. Moreover, treatment with SiO_2_NPs can reduce water stress effects on growth and reproductive structures, thereby avoiding substantial yield loss. According to our obtained data, SiO_2_NPs with a concentration of 90 ppm is the optimal and best concentration for achieving our promising aim. Since this concentration is the highest used, there is still a probability that a higher level leads to more gains, and thereby there is a need for more studies in this regard to continue to explore and examine the alleviate potential of SiO_2_NPs on rice plants growing in water-stressed conditions.

## Figures and Tables

**Table 1:** Physical and chemical analysis of the experimental soil during the 2018 and 2019 seasons.

Seasons	Texture	Sand (%)	Silt (%)	Clay (%)	pH (1:2.5 Soil Extract)	E.C. (dSm^−1^)	Organic Matter %	Available N (ppm)	Available P (ppm)	Available K (ppm)	Available Zn (ppm)	Available Mn (ppm)	Available Fe (ppm)
2018	Clayey	12.8	31.5	55.7	8.33	3.12	1.45	18.4	14.7	322	0.78	3.44	3.12
2019	Clayey	12.0	32.0	56.0	8.40	3.48	1.50	19.1	15.2	347	0.95	3.22	3.70

**Table 2:** Growth characteristics of EHR1 rice variety as affected by water regimes and SiO_2_NPs concentrations during the 2018 and 2019 seasons.

Treatments	Leaf Area Index (LAI)		Dry Matter Production (g/m^2^)	
	2018	2019	2018	2019
Water regimes	–	–	–	–
IR3	7.07 a	6.94 a	1575.1 a	1562.2 a
IR6	6.98 a	6.87 a	1566.0 a	1551.7 a
IR9	5.44 b	5.29 b	1231.2 b	1224.1 b
F test	**	**	**	**
SiO_2_NPs concentrations	–	–	–	–
	–	–	–	–
SiO_2_NPs-0	6.26 d	6.06 d	1415.6 d	1399.5 d
SiO_2_NPs-30	6.41 c	6.28 c	1440.3 c	1428.5 c
SiO_2_NPs-60	6.56 b	6.49 b	1462.2 b	1465.7 b
SiO_2_NPs-90	6.77 a	6.65 a	1501.6 a	1491.6 a
F test	**	**	**	**
IRs X SiO_2_NPs	**	N.S	N.S	**

** and N.S indicate significant at 0.01 level and not significant respectively. IR3, irrigation every 3 days; IR6, irrigation every 6 days; IR9: irrigation every 9 days. SiO_2_NPs-0, without SiO_2_NPs foliar application; SiO_2_NPs-30, foliar application of SiO_2_NPs at 30 ppm; SiO_2_NPs-60, foliar application of SiO_2_NPs at 60 ppm; SiO_2_NPs-90, foliar application of SiO_2_NPs at 90 ppm.

**Table 3:** Influence of the interaction between irrigation treatments (IRs) and SiO_2_NPs concentrations on the leaf area index (LAI) of EHR1 rice variety during the 2018 season.

SiO_2_NPs Concentrations	Water Regimes		
	2018		
	IR3	IR6	IR9
SiO_2_NPs-0	6.71 c	6.75 c	5.32 d
SiO_2_NPs-30	6.96 bc	6.77 bc	5.49 d
SiO_2_NPs-60	7.21 ab	7.06 abc	5.38 d
SiO_2_NPs-90	7.38 a	7.34 a	5.58 d

IR3, irrigation every 3 days; IR6, irrigation every 6 days; IR9: irrigation every 9 days. SiO_2_NPs-0, without SiO_2_NPs foliar application; SiO_2_NPs-30, foliar application of SiO_2_NPs at 30 ppm; SiO_2_NPs-60, foliar application of SiO_2_NPs at 60 ppm; SiO_2_NPs-90, foliar application of SiO_2_NPs at 90 ppm.

**Table 4:** Influence of the interaction between IRs and SiO_2_NPs concentrations on dry matter production of EHR1 rice variety during the 2019 season.

SiO_2_NPs Concentrations	Water Regimes		
	2019		
	IR3	IR6	IR9
SiO_2_NPs-0	1536.2 bc	1518.0 d	1192.3 g
SiO_2_NPs-30	1570.0 b	1546.4 cd	1235.0 f
SiO_2_NPs-60	1580.1 b	1579.2 b	1231.2 f
SiO_2_NPs-90	1623.2 a	1620.4 a	1266.6 e

IR3, irrigation every 3 days; IR6, irrigation every 6 days; IR9: irrigation every 9 days. SiO_2_NPs-0, without SiO_2_NPs foliar application; SiO_2_NPs-30, foliar application of SiO_2_NPs at 30 ppm; SiO_2_NPs-60, foliar application of SiO_2_NPs at 60 ppm; SiO_2_NPs-90, foliar application of SiO_2_NPs at 90 ppm.

**Table 5:** The number of panicles/m^2^, filled grains/panicle, and unfilled grains/panicle of EHR1 rice variety as affected by water regimes and SiO_2_NPs concentrations.

Treatments	The Number of Panicles/m^2^		The Number of Filled Grains/Panicle		The Number of Unfilled Grains/Panicles	
	2018	2019	2018	2019	2018	2019
Water regimes IR3 IR6 IR9 F test	– 616.7 a 612.9 a 447.5 b **	– 613.4 a 609.2 a 438.1 b **	– 154.1 a 151.7 a 118.8 b **	– 150.3 a 146.5 a 113.6 b **	– 6.3 c 7.4 b 15.4 a **	– 7.1 c 8.4 b 17.1 a **
SiO_2_NPs concentrations SiO_2_NPs-0 SiO_2_NPs-30 SiO_2_NPs-60 SiO_2_NPs-90 F test	– 538.0 d 554.7 c 565.3 b 578.1 a **	– 530.4 d 548.4 c 560.3 b 575.2 a **	– 137.3 c 139.3 b 144.1 a 145.4 a **	– 131.7 c 135.8 b 138.0 ab 140.5 a **	– 10.9 a 10.2 b 9.1 c 8.4 d **	– 12.7 a 11.4 b 10.3 c 9.8 c **
IRs X SiO_2_NPs	**	**	N.S	N.S	N.S	N.S

** and N.S indicate significant at 0.01 level and not significant respectively.IR3, irrigation every 3 days; IR6, irrigation every 6 days; IR9: irrigation every 9 days. SiO_2_NPs-0, without SiO_2_NPs foliar application; SiO_2_NPs-30, foliar application of SiO_2_NPs at 30 ppm; SiO_2_NPs-60, foliar application of SiO_2_NPs at 60 ppm; SiO_2_NPs-90, foliar application of SiO_2_NPs at 90 ppm.

**Table 6:** The 1000 grain weight (g), grain yield (t/ha), and biological yield (t/ha) of EHR1 rice variety as affected by water regimes and SiO_2_NPs concentrations during the 2018 and 2019 seasons.

	1000 Grain Weight (g)		Grain Yield (t/ha)		Biological Yield (t/ha)	
Treatments	2018	2019	2018	2019	2018	2019
Water regimes IR3 IR6 IR9 F test	– 26.17 a 26.10 a 24.62 b **	– 26.11 a 26.06 a 24.48 b **	– 12.01 a 11.89 a 8.62 b **	– 11.83 a 11.74 a 8.58 b **	– 27.42 a 27.18 a 21.84 b **	– 27.01 a 26.85 a 21.40 b **
SiO_2_NPs concentrations SiO_2_NPs-0 SiO_2_NPs-30 SiO_2_NPs-60 SiO_2_NPs-90 F test	– 25.34 d 25.54 c 25.68 b 25.95 a **	– 25.27 d 25.37 c 25.62 b 25.93 a **	– 10.41d 10.75 c 10.92 b 11.29 a **	– 10.23 d 10.56 c 10.93 b 11.15 a **	– 24.30 d 25.13 c 25.86 b 26.62 a **	– 23.90 d 24.67 c 25.48 b 26.30 a **
IRs X SiO_2_NPs	**	**	**	**	**	**

** and N.S indicate significant at 0.01 level and not significant respectively. IR3, irrigation every 3 days; IR6, irrigation every 6 days; IR9: irrigation every 9 days. SiO_2_NPs-0, without SiO_2_NPs foliar application; SiO_2_NPs-30, foliar application of SiO_2_NPs at 30 ppm; SiO_2_NPs-60, foliar application of SiO_2_NPs at 60 ppm; SiO_2_NPs-90, foliar application of SiO_2_NPs at 90 ppm.

**Table 7:** Influence of the interaction between water regimes and SiO_2_NPs concentrations on panicles /m^2^ of EHR1 rice variety during the 2018 and 2019 seasons.

SiO_2_NPs Concentrations	Water Regimes					
	2018			2019		
	IR3	IR6	IR9	IR3	IR6	IR9
SiO_2_NPs-0 SiO_2_NPs-30 SiO_2_NPs-60 SiO_2_NPs-90	595.1 c 618.0 b 620.6 b 634.0 a	589.2 d 605.3 c 623.6 ab 633.7 a	430.0 g 441.0 fg 452.3 f 466.6 e	600.0 bc 606.3 b 614.7 ab 632.6 a	584.0 c 605.0 b 619.3 ab 628.6 a	407.3 f 434.0 e 447.1 de 464.3 d

IR3, irrigation every 3 days; IR6, irrigation every 6 days; IR9: irrigation every 9 days. SiO_2_NPs-0, without SiO_2_NPs foliar application; SiO_2_NPs-30, foliar application of SiO_2_NPs at 30 ppm; SiO_2_NPs-60, foliar application of SiO_2_NPs at 60 ppm; SiO_2_NPs-90, foliar application of SiO_2_NPs at 90 ppm.

**Table 8:** Influence of the interaction between water regimes and SiO_2_NPs concentrations on the 1000-grains weight of EHR1 rice variety during the 2018 and 2019 seasons.

SiO_2_NPs Concentrations	Water Regimes					
	2018			2019		
	IR3	IR6	IR9	IR3	IR6	IR9
SiO_2_NPs-0 SiO_2_NPs-30 SiO_2_NPs-60 SiO_2_NPs-90	25.76 d 26.15 bc 26.32 ab 26.46 a	25.90 cd 25.99 cd 26.13 bc 26.37 ab	24.37 f 24.51 f 26.69 f 25.02 e	25.88 bc 25.91 bc 26.25 ab 26.39 a	25.71 c 25.90 bc 26.26 ab 26.38 a	24.22 e 24.30 e 24.37 e 25.03 d

IR3, irrigation every 3 days; IR6, irrigation every 6 days; IR9: irrigation every 9 days. SiO_2_NPs-0, without SiO_2_NPs foliar application; SiO_2_NPs-30, foliar application of SiO_2_NPs at 30 ppm; SiO_2_NPs-60, foliar application of SiO_2_NPs at 60 ppm; SiO_2_NPs-90, foliar application of SiO_2_NPs at 90 ppm.

**Table 9:** Influence of the interaction between water regimes and SiO_2_NPs concentrations on grain yield of EHR1 rice variety during the 2018 and 2019 seasons.

SiO_2_NPs Concentrations	Water Regimes					
	2018			2019		
	IR9	IR6	IR9	IR9	IR6	IR9
SiO_2_NPs-0 SiO_2_NPs-30 SiO_2_NPs-60 SiO_2_NPs-90	11.42 d 11.94 bc 12.21 ab 12.49 a	11.40 d 11.70 cd 12.01 bc 12.41 a	8.35 f 8.56 ef 8.61 e 8.97 e	11.25 e 11.73 c 12.08 ab 12.24 a	11.29 e 11.52 d 11.98 b 12.18 a	8.14 h 8.40 g 8.73 g 9.02 f

IR3, irrigation every 3 days; IR6, irrigation every 6 days; IR9: irrigation every 9 days. SiO_2_NPs-0, without SiO_2_NPs foliar application; SiO_2_NPs-30, foliar application of SiO_2_NPs at 30 ppm; SiO_2_NPs-60, foliar application of SiO_2_NPs at 60 ppm; SiO_2_NPs-90, foliar application of SiO_2_NPs at 90 ppm.

**Table 10:** Influence of the interaction between water regimes and SiO_2_NPs concentrations on the biological yield of EHR1 rice variety during the 2018 and 2019 seasons.

SiO_2_NPs Concentrations	Water Regimes					
	2018			2019		
	IR3	IR6	IR9	IR3	IR6	IR9
SiO_2_NPs-0 SiO_2_NPs-30 SiO_2_NPs-60 SiO_2_NPs-90	26.30 de 27.04 bcd 27.77 b 28.60 a	25.94 e 26.70 cd 27.42 bc 28.67 a	20.71 h 21.67 g 22.40 fg 22.58 f	25.87 d 26.59 c 27.46 b 28.11 a	25.65 d 26.30 c 27.15 b 28.28 a	20.17 h 21.12 g 21.82 f 22.52 e

IR3, irrigation every 3 days; IR6, irrigation every 6 days; IR9: irrigation every 9 days. SiO_2_NPs-0, without SiO_2_NPs foliar application; SiO_2_NPs-30, foliar application of SiO_2_NPs at 30 ppm; SiO_2_NPs-60, foliar application of SiO_2_NPs at 60 ppm; SiO_2_NPs-90, foliar application of SiO_2_NPs at 90 ppm.

**Table 11:** The chlorophyll content is affected by water regimes and SiO_2_NPs concentrations of the EHR1 rice variety during the 2018 and 2019 seasons.

Seasons	Water Regimes				SiO_2_NPs Concentrations					IRs X SiO_2_NPs
	IR3	IR6	IR9	F Test	SiO_2_NPs-0	SiO_2_NPs-30	SiO_2_NPs-60	SiO_2_NPs-90	F Test	F Test
2018	44.67 a	44.32 a	36.68 b	**	40.98 d	41.56 c	42.00 b	43.04 a	**	**
2019	43.87a	43.36 a	36.27 b	**	39.86 d	40.59 c	41.63 b	42.59 a	**	**

** Indicates significant at 0.01 level. IR3, irrigation every 3 days; IR6, irrigation every 6 days; IR9: irrigation every 9 days. SiO_2_NPs-0, without SiO_2_NPs foliar application; SiO_2_NPs-30, foliar application of SiO_2_NPs at 30 ppm; SiO_2_NPs-60, foliar application of SiO_2_NPs at 60 ppm; SiO_2_NPs-90, foliar application of SiO_2_NPs at 90 ppm.

**Table 12:** The chlorophyll content is affected by the interaction between water regimes and SiO_2_NPs concentrations during the 2018 and 2019 seasons.

SiO_2_NPs Applications	Water Regimes					
	2018			2019		
	IR3	IR6	IR9	IR3	IR6	IR9
SiO_2_NPs-0 SiO_2_NPs-30 SiO_2_NPs-60 SiO_2_NPs-90	43.85 cd 44.24 cd 44.76 bc 45.83 a	43.67 d 44.23 cd 44.11 cd 45.35 ab	35.43 f 36.23 f 37.15 e 37.93 e	42.34 d 43.33 cd 44.70 ab 45.11 a	41.95 e 42.70 de 43.91 bc 44.90 a	35.30 h 35.75 gh 36.30 g 37.76 f

IR3, irrigation every 3 days; IR6, irrigation every 6 days; IR9: irrigation every 9 days. SiO_2_NPs-0, without SiO_2_NPs foliar application; SiO_2_NPs-30, foliar application of SiO_2_NPs at 30 ppm; SiO_2_NPs-60, foliar application of SiO_2_NPs at 60 ppm; SiO_2_NPs-90, foliar application of SiO_2_NPs at 90 ppm.

## Data Availability

The data presented in this study are available on request from the corresponding author.
